# Sex-Specific Multiparameter Blood Test for the Early Diagnosis of Alzheimer’s Disease

**DOI:** 10.3390/ijms232415670

**Published:** 2022-12-10

**Authors:** Hyung Joon Cho, Philip Schulz, Lalitha Venkataraman, Richard J. Caselli, Michael R. Sierks

**Affiliations:** 1 Department of Internal Medicine, The University of Arizona College of Medicine—Phoenix, Phoenix, AZ 85004, USA; 2 Chemical Engineering, School for Engineering of Matter, Transport and Energy, Arizona State University, Tempe, AZ 85287, USA; 3 Center for Gene Therapy, Nationwide Children’s Hospital, Columbus, OH 43205, USA; 4 Department of Neurology, Mayo Clinic, Scottsdale, AZ 85259, USA

**Keywords:** Alzheimer’s disease, amyloid-β, tau, TDP-43, longitudinal biomarker, blood-based diagnostic, ELISA, protein variants

## Abstract

Blood-based biomarkers are needed for the early diagnosis of Alzheimer’s disease (AD). We analyzed longitudinal human plasma samples from AD and control cases to identify biomarkers for the early diagnosis of AD. Plasma samples were grouped based on clinical diagnosis at the time of collection: AD, mild cognitive impairment (MCI), and pre-symptomatic (preMCI). Samples were analyzed by ELISA using a panel of reagents against nine different AD-related amyloid-β (Aβ), tau, or TDP-43 variants. Receiver operating characteristic (ROC) curves of different biomarker panels for different diagnostic sample groups were determined. Analysis of all of the samples gave a sensitivity of 92% and specificity of 76% for the diagnosis of AD. Early-stage diagnosis of AD, utilizing only the preMCI and MCI samples, identified 88% of AD cases. Using sex-biased biomarker panels, early diagnosis of AD cases improved to 96%. Using the sex-biased panels, we also identified 6 of the 25 control group cases as being at high risk of AD, which is consistent with what is expected given the advanced age of the control cases. Specific AD-associated protein variants are effective blood-based biomarkers for the early diagnosis of AD. Notably, significant differences were observed in biomarker profiles for the early detection of male and female AD cases.

## 1. Introduction

Biomarkers that can facilitate pre-symptomatic diagnosis of Alzheimer’s disease (AD) and distinguish it from other dementias would be extremely valuable clinical tools. Therapeutic intervention for patients with Alzheimer’s disease (AD) should be most effective if begun early before significant neurological damage has taken place, as suggested by several recent clinical trials targeting Aβ [1,2]. The two most promising fluid biomarkers for AD to date are variants of Aβ and tau, in particular, the 42 amino acid variants of Aβ (Aβ42) and phosphorylated variants of tau [3,4,5,6,7]. However, Aβ and tau levels are not sufficient for a clinical diagnosis of AD, instead positron emission tomography (PET) scans to detect amyloid plaques or tau tangles can be used to confirm a clinical diagnosis. While cerebrospinal fluid (CSF) levels of Aβ42 and phosphorylated tau correlate with AD, these protein variant forms may not represent the best biomarkers for AD as they are also present in cognitively normal individuals and are not the relevant toxic protein species responsible for neurodegeneration in AD. Small soluble oligomeric forms of Aβ and tau are widely but not universally considered to be the relevant toxic protein variants responsible for neurodegeneration. Oligomeric forms of both Aβ and tau, indeed, correlate much better with disease outcomes [8,9] and different oligomeric forms are thought to be responsible for the spread of pathology in the brain [10,11,12]. Different oligomeric variants of both Aβ and tau, therefore, represent promising biomarkers for AD, particularly during early, ideally pre-symptomatic, stages of AD.

Individual AD cases vary considerably and can encompass a spectrum of conditions that often involve pathologies of proteins other than Aβ and tau, including Tar DNA binding protein 43 (TDP-43). Aggregated toxic variants of TDP-43 have been correlated with several neurodegenerative diseases including AD, amyotrophic lateral sclerosis (ALS), and frontotemporal dementia (FTD) [13]. TDP-43 inclusions are present in a subset of AD cases, primarily in limbic regions [14,15] where they can overlap with tau pathology [16] as well as in a significant fraction of all elderly patients [17]. TDP-43 is prone to form aggregate species, where TDP-43 mutations linked to an increased risk of sporadic ALS aggregate more readily [18]. Therefore, small toxic oligomeric variants of Aβ, tau, and TDP-43 can all play critical roles in the onset and progression of AD and related dementias (ADRDs) and all represent promising biomarkers for the early detection of neurodegeneration. Since individual AD cases can express different symptoms and rates of progression, a multiparameter personalized diagnostic approach for AD, similar to the personalized diagnostics now being utilized in the cancer field, could greatly improve the diagnostics and treatment of ADRDs. Individual AD patients will likely have their own distinct pattern of toxic protein variants of Aβ, tau, and TDP-43 and as neurodegeneration progresses and spreads to different brain regions, each patient’s protein variant fingerprint will likely change.

To demonstrate the potential of a multiparameter personalized diagnostic assay for AD, we utilized a panel of nine previously generated and characterized single-chain variable fragments (scFvs) that selectively bind AD-related variants of Aβ, tau, and TDP-43 [19,20,21,22,23,24,25,26,27,28,29,30,31,32] (Table 1). Each scFv also effectively selected AD from age-matched control serum samples [32,33]. Here, the panel of scFvs was used to analyze a set of blinded longitudinal plasma samples obtained from 50 different human volunteers; 25 that converted to AD during the time course of the study, and 25 that remained cognitively normal. Three of the nine scFvs, A4, E1, and C6T, were selected based on specific reactivity with different AD-related variants of Aβ [28,29,30]; A4 and E1 were isolated against different synthetically generated oligomeric Aβ variants [28,29], while C6T was isolated using AD brain-derived oligomeric variants of Aβ [29]. All three protein variants have been detected in human AD brain tissue, CSF, and/or sera/plasma samples [28,29,30,33,34]. Five of the nine scFvs were selected based on selective reactivity toward different AD-related variants of tau [31,32]. The D11C and F9T scFvs were both isolated against synthetically generated toxic trimeric variants of tau but they bind different conformational variants [31]. Both tau variants have been previously detected in human AD brain tissue and plasma samples [31,33]. Three of the tau scFvs, ADTau2, ADTau4, and ADTau6 (previously termed ADT-2, ADT-4, and ADT-6), were isolated against human AD brain-derived tau variants that are also present in human AD plasma samples [32]. These three scFvs against brain-derived tau variants were previously used to discriminate between AD and control samples using the same patient samples as utilized in this study [32]. Here, we utilize data from the three to five individual time points of the AD and control cases obtained with ADTau2, ADTau4, and ADTau6 as part of our multiparameter panel analyses. The final scFv in the panel, AD-TDP3, was isolated against a human ALS brain-derived TDP-43 variant [26,27], but it also has strong reactivity with brain tissue and plasma samples from AD cases [33,34]. In a small pilot study analyzing longitudinal plasma samples with a subset of these scFvs, we showed that blood samples from patients that converted to AD were significantly different from samples of the patients that remained cognitively normal [27,33]. In the pilot study, oligomeric Aβ and TDP-43 variants were present in the earliest available plasma samples of patients that later converted to AD up to seven years prior to the initial diagnosis of MCI, indicating promise for a blood-based pre-symptomatic diagnosis of AD, while oligomeric Aβ and tau variants characterized plasma samples taken after a clinical diagnosis of AD had been made [33]. A recent study showing the presence of Aβ variants in plasma provides further evidence for the promise of blood-based biomarkers that can diagnose and classify AD patients even many years before any clinical symptoms are evident [35]. In the current study, we demonstrate that a panel of scFvs that selectively bind AD-related protein variants of Aβ, tau, and TDP-43 is a very effective tool to identify novel pre-symptomatic blood-based biomarkers of AD and that there is a sex bias in these blood-based protein variant biomarkers.

## 2. Results

### 2.1. Protein Variant Profiles (AD vs. Controls)

**Figure 1:**
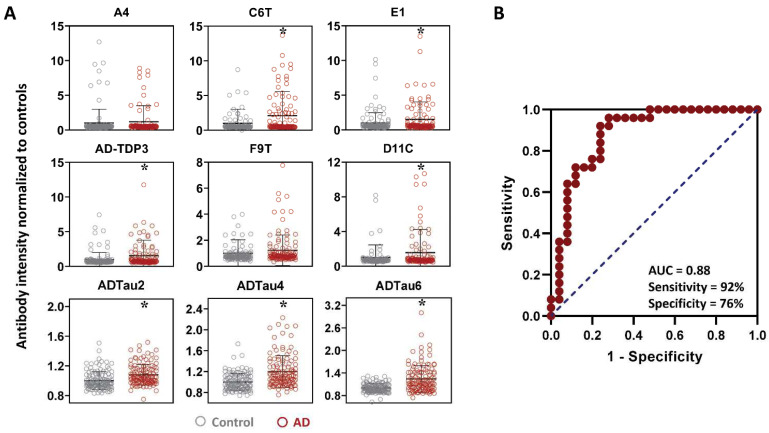
Analysis of all longitudinal AD and control plasma samples. (**A**) Protein variants levels of all the cases assessed using a capture ELISA and a panel of 9 scFvs. Significant differences were obtained with 7 of the 9 scFvs: 2 Aβ scFvs (C6T, E1), TDP-43 scFv (AD-TDP3), and 4 tau scFvs (D11C, ADTau2, ADTau4, and ADTau6). Data represent mean average score and standard deviation with statistical significance by ANOVA post hoc LSD * *p* < 0.05. (**B**) ROC (receiver operating characteristic) curve for 7 statistically significant scFvs using averaged time point ELISA values for each 25 AD and 25 control cases. From the AUC (0.88), an optimal cutoff threshold (11.00) was calculated by maximum Youden J value (0.68; sensitivity + specificity − 1), with a sensitivity of 92% and specificity 76%.

**Figure 2:**
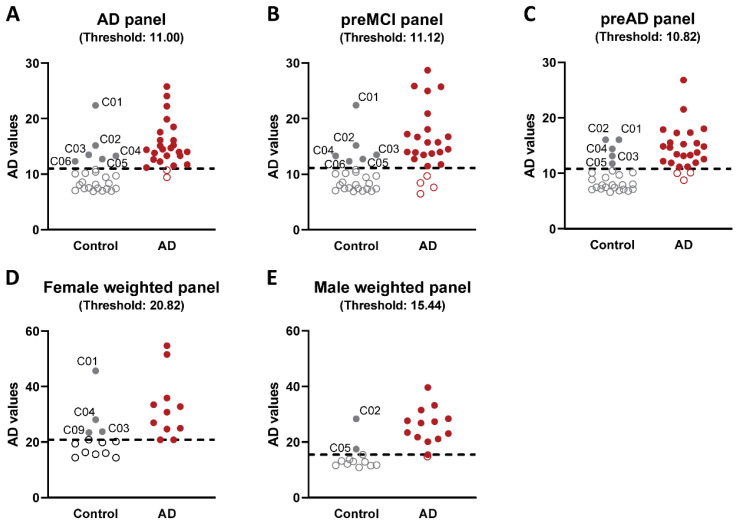
ROC ranking of control and AD cases with different antibody panels. Plot of cumulative biomarker levels of various different plasma samples from AD and control cases analyzed with different biomarker panels. Cutoff thresholds from ROC analyses shown as dotted lines. (**A**) Plot of cumulative biomarker levels of all samples from 25 control and 25 AD cases using the AD biomarker panel (Aβ—C6T, E1; tau—D11C, ADTau2, ADTau4, ADTau6; TDP-43—AD-TDP3). The panel detected 23 out of 25 AD cases and flagged 6 of the 25 controls (filled circles). (**B**) Similar plot of preMCI only samples from 24 AD cases and all samples from 19 unflagged control cases using the same biomarker panel in (**A**). The panel detected 20 out of 24 AD cases and flagged 6 out of 25 controls (filled circles). (**C**) Similar plot of preAD samples only from 25 AD cases and all samples from 20 unflagged control cases using the preAD biomarker panel (Aβ—C6T; tau—D11C, F9T, ADTau2, ADTau4, ADTau6; TDP-43—AD-TDP3). The panel detected 22 out of 25 AD cases and flagged 5 out of 25 controls (filled circles). (**D**) Similar plot of preAD samples only from female AD cases and samples from female unflagged control cases using the female-biased biomarker panel (Aβ—C6T, **E1**; tau—**D11C, F9T, ADTau2, ADTau4, ADTau6**; TDP-43—**AD-TDP3**) (biomarkers in **bold** were weighted double). The panel detected 11 out of 11 female AD cases and flagged 4 out of 13 female control cases (filled circles). (**E**) Similar plot of preAD samples only from male AD cases and samples from male unflagged control cases using the male-biased biomarker panel (Aβ—**C6T**, tau—D11C, F9T, **ADTau2, ADTau4, ADTau6**; TDP-43—AD-TDP3) (biomarkers in **bold** were weighted double). The panel detected 13 out of 14 male AD cases and flagged 2 out of 12 male control cases (filled circles).

All of the plasma samples from the control and AD cases were analyzed for reactivity with a panel of 9 scFvs; three that bind different Aβ variants (A4, C6T, and E1), five that bind different tau variants (D11C, F9T, ADTau2, ADTau4, and ADTau6) and one that binds a TDP-43 variant (AD-TDP3) (Table 1). Seven of the nine scFvs utilized showed statistically significant differences in reactivity between the plasma samples from the AD cases compared to the plasma samples from the control cases, including two Aβ variants (C6T and E1), four tau variants (D11C, ADTau2, ADTau4, and ADTau6) and the TDP-43 variant (AD-TDP3) (Figure 1A). Based on ROC analysis with an area under curve (AUC) of 0.88 and a maximum Youden J index of 0.68, the panel of seven statistically significant scFvs demonstrated a sensitivity of 92% and a specificity of 76% (Figure 1B). A plot of the composite biomarker scores for the 25 control cases and 25 AD cases using the ROC cutoff value showed two AD cases below the cutoff and six flagged control cases above the cutoff (Figure 2A). This 7 scFvs biomarker panel represents a promising set of reagents from which to develop a multiparameter biomarker panel for the early diagnosis of AD.

### 2.2. Analysis of Flagged Control Cases as Potential Pre-Symptomatic AD Cases

An inherent difficulty in identifying early-stage biomarkers for AD is that a significant percentage of cognitively normal elderly adults are likely to be pre-symptomatic for ADRDs. The average age of the control group at the end of this study was almost 86 years (Table 2), and since approximately 1/3 of adults of age 85 and over are expected to develop AD [36], a significant percentage of the control cases were anticipated to actually be pre-symptomatic for ADRDs. Numerous previous studies have validated the incidence of AD pathology or underdiagnosis of dementia in cognitively normal elderly patients, indicating that between 25–40% of cognitively normal elderly patients likely have undiagnosed dementia [17,37,38,39,40,41]. Based on these literature studies, around 5–10 of the 25 control cases used in our study were expected to be pre-symptomatic ADRD cases. Including these potentially early-stage pre-symptomatic ADRD cases in the cognitively normal control data set could mask the identification of promising early-stage AD biomarkers.

We reasoned that the six control cases flagged as AD likely comprise some or most of the roughly 20–40% of undiagnosed pre-symptomatic AD cases that could be expected among the 25 control cases in this study based on the ages and ApoE allele distribution (Supplemental Table S1). In order to identify the control cases that are most likely to have underlying early-stage AD pathology, the components of early-stage AD protein variant profiles using our scFv panel were then determined. Samples from the AD cases included samples taken at time points starting when each participant was clinically cognitively normal and ending after each patient was clinically diagnosed as AD. The plasma samples collected from the AD cases were separated into three different groups; (1) samples collected from patients prior to any clinical diagnosis of MCI (labeled as preMCI), (2) samples collected after diagnosis of MCI but before diagnosis of AD (labeled as MCI), and (3) samples collected after diagnosis of AD (labeled as AD). Nine samples taken from AD patients without a clinical diagnosis of cognitively normality, MCI, or AD at the time of collection were excluded. We hypothesized that a good early-stage AD biomarker panel would identify a high percentage of the preMCI samples from the AD cases and some of the samples from the six control cases flagged as potential pre-symptomatic AD cases, but none of the samples from the remaining cognitively normal controls did.

**Figure 3:**
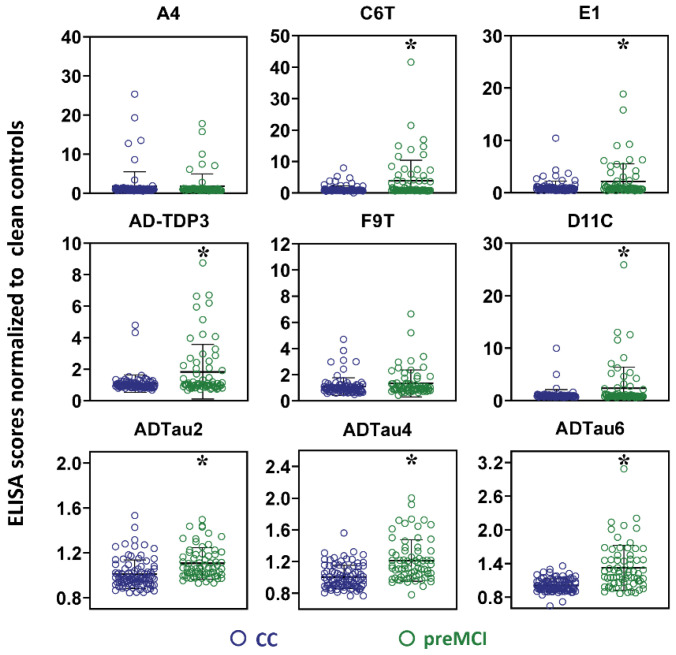
Comparison of plasma samples from preMCI cases with 19 unflagged control cases. Levels of nine different protein variants measured by capture ELISA in preMCI samples from AD cases and unflagged control samples. Significant differences were obtained with seven of the nine scFvs: Aβ (C6T, E1), TDP-43 (AD-TDP3), and tau (D11C, ADTau2, ADTau4, and ADTau6). Data represent mean average score and standard deviation with statistical significance by ANOVA post hoc LSD * *p* < 0.05. CC denotes clean controls as unflagged control cases.

The protein variant panel levels of the 19 unflagged control cases were compared to the protein variant panel levels of the preMCI samples from the 24 AD cases (one AD case did not have any preMCI plasma samples). The same 7 out of 9 scFvs showed statistically significant differences between the preMCI samples from the AD cases and the samples from the 19 unflagged control cases (all scFvs except A4 and F9T) (Figure 3). This panel of seven scFvs was termed the preMCI biomarker panel (Table 3).

**Figure 4:**
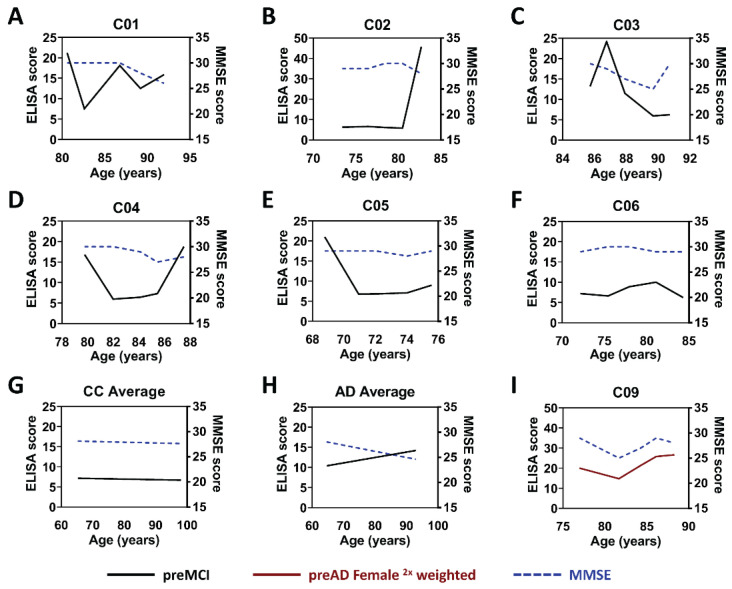
Time course of biomarker reactivity and MMSE scores for six flagged control cases. The preMCI biomarker panel scores (black) and MMSE scores (blue) were plotted as a function of time for the following cases: (**A**–**F**) the six flagged control cases; C01–C06, (**G**) composite profile for the 20 unflagged control cases, (**H**) composite profile for the 25 AD cases, (**I**) control case C09 flagged using the female-biased biomarker panel. Five of the six flagged control cases (C01 through C05) (**A**–**E**) show an increase in biomarker panel reactivity and decrease in MMSE scores, particularly at the later time points resembling the composite AD plots (**H**). The C06 case did not show an increase in biomarker scores or decrease in MMSE score (**F**) and closely resembled the composite control plots (**G**). The results from the female control case (C09; (**I**)) flagged by the female-biased preAD biomarker panel suggest this individual case may be at high risk of AD. CC denotes clean controls as unflagged control cases.

We analyzed the protein variant profiles of the six original flagged control samples obtained with the preMCI panel to determine which ones were likely to be pre-symptomatic AD cases. The protein variant profiles and the mini-mental state examination (MMSE) scores were plotted as a function of age for each of these flagged controls (Figure 4A–F), along with a composite plot for the remaining 19 unflagged control cases (Figure 4G) and 25 AD cases (Figure 4H). Five of the six initially flagged control samples (C01, C02, C03, C04, and C05) (Figure 4A–E) showed increases in the levels of the preMCI biomarker panel, especially in the samples collected from the last two time points available, while the sixth (C06) (Figure 4F) did not show significant changes over collecting time points. The composite control cases (Figure 4G) showed essentially no change in the biomarker panel levels with age, while the composite AD cases (Figure 4H) showed a significant increase in biomarker levels with age. Four of the six flagged controls (C01, C02, C03, and C04) (Figure 4A–D) also showed a decrease in MMSE score with age, particularly in the last time points, while two of the flagged controls (C05 and C06) (Figure 4E,F) remained constant. The MMSE scores for the composite control cases were essentially constant with age (Figure 4G) while the MMSE scores for the composite AD cases decreased with age (Figure 4H). The high reactivity of preMCI panel biomarkers in the last two time points for the flagged control cases C01 through C05, along with a decrease in MMSE score in cases C01 through C04, provided additional evidence that these five flagged controls (C01 through C05) were likely to be pre-symptomatic AD cases. The sixth flagged control case (C06) did not show reactivity with the six statistically significant preMCI biomarker panel or a decrease in MMSE at any of the time points, and, therefore, this control case was not considered to be at increased risk of AD and was reincluded into the cognitively normal control.

### 2.3. Analysis of Samples from AD Cases before Clinical Diagnosis of AD

**Figure 5:**
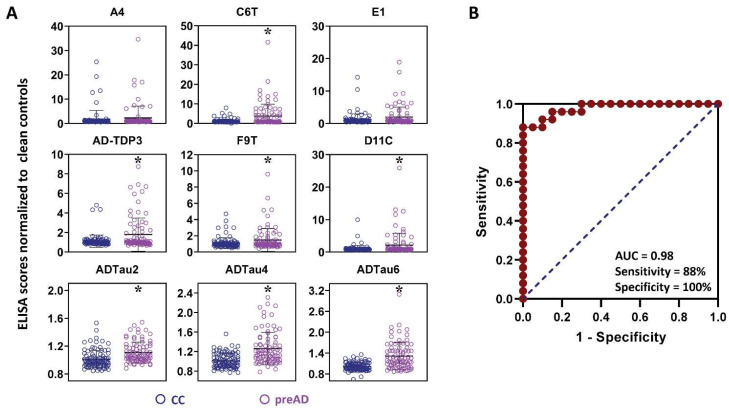
Comparison of plasma samples from preAD cases with 20 unflagged control cases (**A**) Levels of 9 different protein variants measured in preAD and 20 unflagged control samples. Significant differences were obtained with seven scFvs including: Aβ (C6T), TDP-43 (AD-TDP3), and tau (D11C, F9T, ADTau2, ADTau4, and ADTau6). Data represent mean average score and standard deviation with statistical significance by ANOVA post hoc LSD * *p* < 0.05. (**B**) ROC curve for 7 statistically significant scFvs (preAD screening panel) using averaged time point ELISA values for 25 preAD cases with AD and 20 unflagged control cases. From the AUC (0.98), an optimal cutoff threshold (10.82) was calculated by maximum Youden J value (0.88), with a sensitivity of 88% and specificity of 100%. CC denotes clean controls as unflagged control cases.

We expanded our analysis to include samples taken after a clinical diagnosis of MCI but before a diagnosis of AD along with the preMCI samples, labeling these combined time points as “preAD”. Seven of the nine scFvs (C6T, AD-TDP3, F9T, D11C, ADTau2, ADTau4, and ADTau6) demonstrated statistically significant differences between the 25 preAD sample set and the 20 control cases (minus the 5 flagged controls) (Figure 5A). This panel of seven statistically significant scFvs was termed the “preAD panel” (Table 3). The preAD panel included the F9T scFv against a trimeric tau variant [31] which did not have statistical significance in the preMCI panel. Significantly, the preMCI panel includes four scFvs, C6T, AD-TDP3, ADTau4, and ADTau6, which were previously shown to have high reactivity with preMCI blood samples from AD patients [32,33], while the preAD panel includes the F9T scFv, which was previously identified as having high reactivity with AD samples taken from MCI but not preMCI time points [33]. ROC analysis on the preAD (preMCI/MCI) samples from the 25 AD cases and the 20 control cases (minus the 5 flagged controls) performed using the preAD biomarker panel gave an AUC of 0.98, sensitivity of 88%, and specificity of 100%. (Figure 5B). Five of the six control cases (C01–C05) which were previously flagged as AD in the preMCI study (Figure 2B) were again flagged using the preAD biomarker panel (Figure 2C). The sixth previously flagged control case C06 which was reincluded into the control cohort was not flagged by the preAD panel.

### 2.4. Sex Differences in Protein Variant Biomarkers

**Figure 6:**
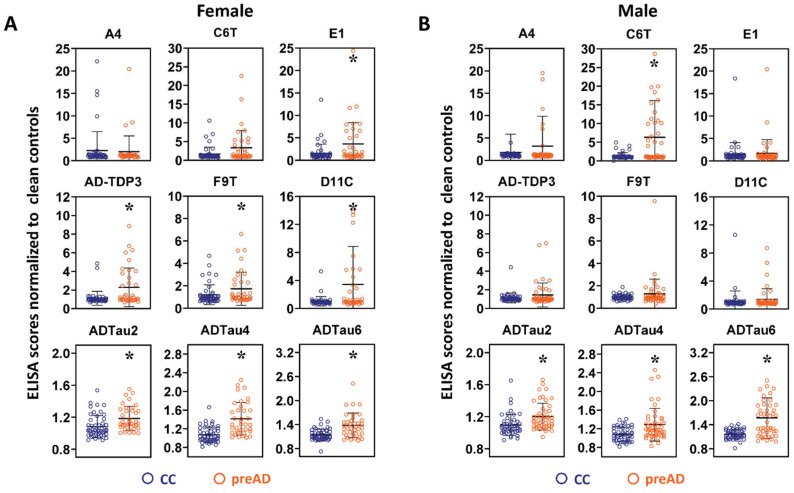
Sex differences in preAD cases and unflagged control plasma sample significance. Levels of 9 different protein variants measured in preAD (preMCI and MCI) time points in 25 AD cases and 20 unflagged control cases separated by sex. (**A**) The preAD samples from female AD cases showed significant differences between female control cases with seven of nine scFvs: Aβ (E1), TDP-43 (AD-TDP3), and tau (F9T, D11C, ADTau2, ADTau4, and ADTau6). (**B**) The preAD samples from male AD cases showed significant differences between male control cases with four of nine scFvs: Aβ (C6T) and tau (ADTau2, ADTau4, and ADTau6). Data represent mean average score and standard deviation with statistical significance by ANOVA post hoc LSD * *p* < 0.05. CC denotes clean controls as unflagged control cases.

The protein variant levels in female and male cases were separately analyzed. Comparison of the preAD samples from female AD cases to the female unflagged control samples identified seven scFvs (E1, AD-TDP3, F9T, D11C, ADTau2, ADTau4, and ADTau6) with significant differences (Figure 6A). Similar comparison of preAD samples from male AD cases to male unflagged control samples detected four scFvs (C6T, ADTau2, ADTau4, and ADTau6) with significant differences (Figure 6B). In total, five of the scFvs differed between females and males, with E1, AD-TDP3, F9T, and D11C all selecting the female cases and C6T selecting the male cases. ROC curves with the preAD biomarker set were re-evaluated to reflect differences in the biomarker preferences based on sex. For analysis of the female cases, we increased the weight of the female selective biomarkers by a factor of two (E1, AD-TDP3, F9T, D11C, ADTau2, ADTau4, and ADTau6) (Table 3), and similarly for the male cases increased the weight of the male selective biomarkers by a factor of two (C6T, ADTau2, ADTau4, and ADTau6) (Table 3). ROC analysis of the preMCI/MCI samples from the female AD (11 of 25) and control cases (13 of 25) identified all 11 AD cases and flagged 4 of 13 control cases, the identical 3 controls previously flagged, plus 1 additional female control case (C09) (Figure 2D). Analysis of the male AD and control cases detected 13 of 14 AD cases and flagged the same 2 control cases (Figure 2E). Further analysis of the additional female control case flagged by the female preAD panel (C09) indicates that this case also has an increase in protein variant levels at the later time points and instability in MMSE scores (Figure 4I) suggesting that this control case may also be at high risk of AD. Interestingly, four of the six control cases flagged as early AD are females, which is consistent with reports that females may have a higher cognitive reserve which can result in a later diagnosis of dementia [42]. With this very simple preliminary 2× weighting of the biomarker panels based on sex, a sensitivity of 96% and specificity between 76 and 100% was obtained, where specificity ultimately depends on how many of the flagged control cases are correctly identified as pre-symptomatic AD cases. Since five of the six flagged control cases have increasing biomarker levels with decreasing or erratic MMSE scores at the most recent time points and the five undiagnosed dementia cases are at the low end of the number of cases expected for this control group, we expect that several, if not most, of these cases are pre-symptomatic AD cases.

### 2.5. Early Diagnosis Time Frame

To estimate how effective the biomarker panels are for early diagnosis of AD, individual time point samples from all the AD cases were analyzed to determine when the first diagnosis of AD was made (Table 4). Using the preAD biomarker panel to analyze the preMCI/MCI samples, 22 of 25 AD cases were detected as AD, where 14 of 25 were identified by the earliest time point sample available. For the 22 identified AD cases using the preAD biomarker panel, a diagnosis of AD could be made on average 6.0 years before a clinical diagnosis of AD (Table 4). Analyzing the female and male AD preMCI/MCI samples separately using the respective female and male preAD biomarker panels, all 11 female AD cases were identified as AD, 7 by the earliest available sample, and 13 out of 14 male AD cases were detected as AD, 9 by the earliest available sample. For the 24 detected AD cases using the sex-specific biomarker panels, a diagnosis of AD could be made on average 6.3 years before a clinical diagnosis of AD. Since the majority of the AD cases detected using the sex-specific biomarker panels (16/25) were identified using the first available time point, we are optimistic that an AD diagnosis can be made significantly earlier.

## 3. Discussion

AD is a progressive neurodegenerative disorder [43,44] associated with the generation of protein variants of Aβ, tau, and TDP-43 [13,34,45,46,47,48,49]. In this blinded longitudinal study, we demonstrated that the selected disease-specific conformational variants of these three proteins are very promising blood-based biomarkers for early, even pre-symptomatic, diagnosis of AD cases. In a blinded study of 25 AD and 25 control cases, we could detect 88% of AD cases before a clinical diagnosis of AD (Figure 5). In addition, significant differences in the biomarker profiles were observed between female AD cases and male AD cases (Figure 6). By analyzing the data using biomarker panels tailored for these sex differences, a significantly higher percentage of AD cases (96%—all but one male AD case) were identified, where most of these cases could be diagnosed using the earliest available plasma samples (16 out of 25), including 15 out of 24 cases diagnosed pre-symptomatically. Quite notably, diagnosis of AD using the sex-biased biomarker panels detected a higher percentage of AD cases (96% vs. 88%) at an earlier time point (6.3 vs. 6.0 years before clinical AD diagnosis) (Table 4). Since the average age of the control group at the end of the study was around 86 years, it is expected that between 20 and 40% of the 25 control cases should be pre-symptomatic for different neurodegenerative diseases [17,37,38,39,40,41]. We identified six control cases using the sex-biased biomarker panels that had protein variant profiles similar to the early-stage pre-symptomatic AD cases (Figure 2D,E). Five of these flagged control cases demonstrated a decline or erratic changes in MMSE scores with age and an increase in biomarker profiles in the most recent blood samples (Figure 4). While the sample sizes of the split male and female cohorts are rather small, the sex differences are significant and indicate that sex, along with genotypes such as ApoE, should play an important factor in developing precision diagnostics for AD. Unfortunately, follow-up cognitive testing of the 25 control cases is not available to validate the outcomes of the flagged controls; however, we intend to test a larger set of pathologically confirmed longitudinal AD cases to further support the validity of these biomarkers as diagnostics reagents for AD and to better define the composition of the female and male biomarker panels. Moreover, additional scFvs that selectively target protein variants implicated in other neurodegenerative diseases including Parkinson’s disease, ALS, and frontotemporal lobar dementia are also available [20,21,27,50]. Since many neurodegenerative disease cases exhibit overlapping pathologies, these results suggest the possibility for developing blood-based precision diagnostics and accompanying therapeutic treatment plans that are personalized to each patient’s individual proteinopathy pattern.

## 4. Material and Methods

### 4.1. Study Population

The sets of longitudinal plasma samples from 50 different human subjects with 3–5 time points per subject were obtained from Mayo Clinic collections by Dr. Steven Younkin and provided with the assistance of Dr. Terrone Rosenberry at the Mayo Clinic College of Medicine in Jacksonville, FL, USA. The samples were selected to provide similar age and sex characteristics (Table 2) and to be representative of ApoE allele distribution (Supplementary Table S1). Of the cases provided, 25 converted to AD while 25 remained cognitively normal based on clinical diagnoses. Samples were randomized and blinded, and their identification was only revealed after completing ELISA analyses.

Initial screening included self-report, the Folstein mini-mental state examination (MMSE) [51], and the Hamilton depression scale [52]. Further support was then provided by an extensive battery of neuropsychological tests [53]. Self and informant responses were obtained on the clinical dementia rating scale [54].

Patients were subsequently evaluated by a behavioral neurologist specializing in dementia-related disorders. All patients had a complete neurological examination that included mental status testing, brain imaging (MRI), and a standard battery of laboratory tests that included thyroid, vitamin B12, chemistries, and blood counts. All patients underwent a standardized neuropsychological battery of tests (uniform data set) that encompassed memory, executive, language, and visuospatial skills as well as behavioral domains including depression and anxiety. Diagnosis of MCI [55] and AD [56] fulfilled published clinical criteria.

### 4.2. Capture ScFvs

All nine scFvs were produced and purified as previously described [26,34,57] for use as capture antibodies in the sandwich ELISA.

### 4.3. Detection Phages

Pan-specific scFvs reactive with both diseased and non-diseased variants of Aβ (H1v2), tau (TauM1), and TDP-43 (TDPM1) were previously generated and characterized [21,23,33,34,58]. The detection scFvs were displayed on a M13 bacteriophage particle, then surface carboxyl groups were biotinylated to increase the detection signal as previously described [57].

### 4.4. Sandwich ELISA

The sandwich ELISA using the phage particles for detection was used as described previously [32,34,50,57,59]. Briefly, the capture scFv was immobilized to the wells of a high-binding 96-well ELISA plate (Corning, Corning, NY, USA) and any unbound sites were blocked with 2% milk in phosphate-buffered saline (PBS). Plasma samples were diluted 1/100 (*v*/*v*) in PBS and then 100 μL of each sample was added to individual wells of a 96-well ELISA plate. All of the samples were analyzed in triplicate. Following incubation for 1 h at 37 °C, 200 ng/mL of the respective biotinylated detection phages were added. Any bound phages were identified using avidin conjugated with HRP (Sigma-Aldrich, St. Louis, MO, USA) to label the biotinylated phage coat proteins. Following the addition of the SuperSignal ELISA Femto Maximum Sensitivity Substrate (Thermo Scientific-Pierce, Rockford, IL, USA), signal intensities were quantified using a Wallac Victor^2^ microplate reader. After each incubation step, the wells were washed 3–4 times with 0.1% PBS-Tween20 to reduce non-specific binding. Known positive and negative controls were included on each ELISA plate. Collective data obtained using the ADTau2, ADTau4, and ADTau6 scFvs to analyze the AD and control samples were previously reported [32], and data obtained with the individual time point samples in the biomarker studies were utilized in this blinded longitudinal study.

### 4.5. Statistical Analysis

All sample signal intensities were normalized to calibration samples (PBS) on each plate and plasma control samples were normalized for consistency across ELISA plates. The plasma samples were all prepared, stored, and analyzed using the same protocols to ensure consistency of the data. Analyses performed with only unflagged control samples were normalized to the respective number of unflagged control samples for each case. ANOVA and Fisher LSD post hoc tests were completed with IBM SPSS 27 or the Agricolae package in R statistical language, with significance based on values *p* < 0.05 [60].

Receiver operating characteristic (ROC) curves and statistics were calculated in SPSS, with specificity and sensitivity values based on optimized threshold cutoffs using the maximized Youden J index (sensitivity + specificity − 1). The appropriate samples for each AD or control case were analyzed to obtain the composite value for each scFv with each case. Analysis was performed on different AD sample subsets, including “AD” which included all samples (n = 25); “preMCI” which included only pre-symptomatic samples taken before a clinical diagnosis of MCI (n = 24); and “preAD” which included all samples taken before a clinical diagnosis of AD (including preMCI and MCI) (n = 25). Analysis of control cases included samples from all time points. We utilized two different control groups for ROC analyses, either containing all control cases (n = 25), or just clean controls (CC) which excluded flagged control cases based on the biomarker profiles obtained in the initial total AD analysis.

## Figures and Tables

**Table 1:** List of scFvs and their target antigens.

scFv	Target Antigen Description
**Aβ variants**	
A4	Synthetically generated oligomeric Aβ
E1	Synthetically generated oligomeric Aβ
C6T	Human AD brain-derived oligomeric Aβ
**Tau variants**	
F9T	Synthetically generated trimeric tau variant
D11C	Synthetically generated trimeric tau variant
ADTau2	Human AD brain-derived tau variant
ADTau4	Human AD brain-derived tau variant
ADTau6	Human AD brain-derived tau variant
**TDP-43 variants**	
AD-TDP3	Human AD and ALS brain-derived TDP-43 variant

**Table 2:** Description of patient population.

	Control	AD
Patients (n)	25	25
Average age (years)	82.1 ± 7.7	80.5 ± 6.1
Average age at final draw (years)	85.9 ± 7.5	83.9 ± 5.4
Sex (n)	M (12), F (13)	M (14), F (11)
Average Participation span (years)	7.7	7.3
Average MMSE *	28.1 ± 1.83	26.1 ± 2.8

* MMSE: mini-mental state examination.

**Table 3:** Composition of biomarker panels.

Biomarker Panel	scFvs in Panel
AD/preMCI	C6T, E1, D11C, ADTau2, ADTau4, ADTau6, AD-TDP3
preAD	C6T, D11C, F9T, ADTau2, ADTau4, ADTau6, AD-TDP3
Female preAD	C6T, **E1**, **D11C**, **F9T**, **ADTau2**, **ADTau4**, **ADTau6**, **AD-TDP3**
Male preAD	**C6T**, D11C, F9T, **ADTau2**, **ADTau4**, **ADTau6**, AD-TDP3

scFvs in **bold print** were weighted double for the respective panel.

**Table 4:** Early detection of AD.

Description	Cases Detected /Total Cases	Average Years before Clinical AD Diagnosis
preAD biomarker panel		
Diagnosis of AD cases from preMCI/MCI samples	22/25	6.0
Detection in 1st available preMCI/MCI sample	14/25	6.7
Diagnosis of AD cases from preMCI samples	19/24	6.2
Detection in 1st available preMCI sample	13/24	6.8
Female-biased biomarker panel		
Diagnosis of female AD cases from preMCI/MCI samples	11/11	6.5
Detected in 1st available female preMCI/MCI sample	7/11	7.1
Diagnosis of female AD cases from preMCI samples	10/11	6.5
Detected in 1st available female preMCI sample	7/11	7.1
Male-biased biomarker panel		
Diagnosis of male AD cases from preMCI/MCI samples	13/14	6.1
Detection in 1st available male preMCI/MCI sample	9/14	6.6
Diagnosis of male AD cases from preMCI samples	11/13	6.6
Detection in 1st available male preMCI sample	8/13	6.8
Combined female/male biomarker panel data		
Diagnosis of female and male AD cases from preMCI/MCI samples	24/25	6.3
Combined female and male detection in 1st available preMCI/MCI sample	16/25	6.8
Diagnosis of female and male AD cases from preMCI samples	21/24	6.5
Combined female and male detection in 1st available preMCI sample	15/24	7.0

## Data Availability

Data are available upon reasonable request from the corresponding author.

## Supplementary Materials

The supporting information can be downloaded at: https://www.mdpi.com/article/10.3390/ijms232415670/s1.
