# Supplementary material for: Sex-Specific Multiparameter Blood Test for the Early Diagnosis of Alzheimer’s Disease
Source: Int J Mol Sci. 2022 Dec 10;23(24):15670. doi: 10.3390/ijms232415670 (PMC9779188; doi:10.3390/ijms232415670)
Supplement: Supplementary file 1 [file ijms-23-15670-s001.zip › ijms-2015015-supplementary.pdf]

International Journal of
Molecular Sciences

MDPI

Article

Sex-Specific Multiparameter Blood Test for the Early Diagnosis of Alzheimer's Disease

Supplemental Table S1: Description of AD and Control Samples

	Control (n = 122)	AD (n = 119)
Diagnosis	Number of samples (average age in years)	Number of samples (average age in years)
Cognitively normal	122 (82.1 ± 7.7)	68 (79.4 ± 5.7)
MCI	-	17 (80.9 ± 6.6)
AD	-	25 (83.9 ± 5.4)
No diagnosis	-	9 (78.5 ± 6.75)
ApoE genotypes (n)	2/2 (1), 2/3 (1), 2/4 (3), 3/3 (16), 3/4 (3), 4/4 (1)	3/3 (11), 3/4 (13), n/a (1)
